# The long noncoding RNA MEG3 regulates Ras-MAPK pathway through RASA1 in trophoblast and is associated with unexplained recurrent spontaneous abortion

**DOI:** 10.1186/s10020-021-00337-9

**Published:** 2021-07-08

**Authors:** Jun Zhang, Xinqiong Liu, Yali Gao

**Affiliations:** 1 Department of Obstetrics and Gynecology, Shenzhen People’s Hospital (The Second Clinical Medical College, Jinan University), Shenzhen, 518020 People’s Republic of China; 2 Department of Ophthalmology, Shenzhen People’s Hospital (The Second Clinical Medical College, Jinan University), Shenzhen, 518020 People’s Republic of China

**Keywords:** URSA, MEG3, RASA1, RAS-MAPK pathway

## Abstract

**Background:**

Maternally Expressed Gene 3 (*MEG3*) is expressed at low levels in placental villi during preeclampsia; however, its roles in unexplained recurrent spontaneous abortion (URSA) remain unclear. In this study, we aimed to explore the relationship between *MEG3* and URSA.

**Methods:**

The differentially expressed lncRNAs (*MEG3*) and its downstream genes (*RASA1*) were identified using bioinformatics analysis of Genomic Spatial Event (GSE) database. The expression levels of *MEG3* in embryonic villis (with gestational ages of 49–63 days) and primary trophoblasts were determined using quantitative RT-PCR assay. A mouse model of Embryo implantation, Cell Counting Kit-8 (CCK-8), flow cytometry, and Transwell migration assays were performed to determine the implantation, proliferative, apoptotic, and invasive capacities of trophoblast. The level of phosphorylated core proteins in the RAS-MAPK pathway were analyzed using Western blot assay. The mechanisms of *MEG3* in the regulation of *RASA1* were studied by RNA pulldown, RNA immunoprecipitation (RIP), DNA pulldown, and chromatin immunoprecipitation (ChIP) assays.

**Results:**

*MEG3* had a low expression level in embryonic villis of 102 URSA patients compared with those of 102 normal pregnant women. MEG3 could promote proliferation and invasion, inhibit the apoptosis of primary trophoblast of URSA patients (PT-U cells), as well as promote embryo implantation of mouse. Besides, MEG3 also promoted the phosphorylation of rapidly accelerated fibrosarcoma (Raf), mitogen-activated protein kinase kinase (MEK), and extracellular-signal-regulated kinase (ERK) proteins. The results of RNA pull down and RIP assays showed that MEG3 bound with the enhancer of zeste homolog 2 (EZH2). The DNA pulldown assay revealed that MEG3 could bind to the promoter sequence of the RAS P21 Protein Activator 1 (*RASA1*) gene. Further, the ChIP assay showed that MEG3 promoted the binding of EZH2 to the promoter region of the *RASA1* gene.

**Conclusions:**

The inactivation of MEG3 in embryonic villi association with URSA; MEG3 inhibited the expression of *RASA1* by mediating the histone methylation of the promoter of *RASA1* gene by EZH2, thereby activating the RAS-MAPK pathway and enhancing the proliferative and invasive capacities of trophoblasts.

**Supplementary Information:**

The online version contains supplementary material available at 10.1186/s10020-021-00337-9.

## Introduction

Recurrent spontaneous abortion (RSA) refers to three or more consecutive spontaneous abortions with multiple complicated etiologies, half of which are unclear and thus, termed as unexplained recurrent spontaneous abortion (URSA). RSA with a known etiology can be treated by targeting underlying causes via various approaches, such as surgeries and drugs, but the diagnosis and treatment of URSA patients remain widely debatable (Ewington et al. 2019). Hence, it is necessary to explore the molecular mechanism underlying the onset and progression of URSA, to provide new ideas and basis for studying the pathogenesis and clinical treatment of URSA.

Long noncoding RNAs (lncRNAs) are functional RNAs that do not encode for proteins but can regulate the expressions of protein-coding genes and protein activities at various molecular levels, including transcriptional, post-transcriptional, post-translational modification, and epigenetic levels. lncRNAs play critical regulatory roles in the growth and development of cells, metabolism, as well as the onset and progression of diseases (Guo 2018; Meng et al. 2019; Li, 2019). In recent years, studies on lncRNA expression profiles in placental villi from patients with RSA have revealed dysregulated expression of various lncRNAs involved in infections, inflammation, immunity, and apoptosis, thereby suggesting that lncRNAs are closely related to the onset and progression of RSA (Wang 2014; Wang et al. 2016).

The transcript of maternally expressed gene 3 (*MEG3*) is an lncRNA that does not encode for protein and has regulatory effect on cell function (Zhang, 2018; Tang et al. 2020; Chanda 2018). Recent studies have shown that the abnormal expression of *MEG3* in placental villi is closely associated with the preeclampsia and MEG3 can regulate the proliferative and invasive capacities of trophoblasts (Zhang 2015; Yu 2018). However, the relationship between MEG3 and URSA remains unclear.

In this study, we identified *MEG3* and its downstream target gene *RASA1* from the GSE database. We also found that *MEG3* had a low expression level in embryonic villis from URSA patients (with gestational ages of 49–63 days) compared with those of normal pregnant women. Functional studies revealed that the low expression of *MEG3* could inhibit the implantation (mouse model), proliferation and invasion, as well as, promote the apoptosis of primary trophoblast of URSA patients (PT-U cells). Finally, we demonstrated that MEG3 promotes the histone methylation and silences the expression of *RASA1* gene by recruiting EZH2 to the promoter of the *RASA1* gene, which encodes Ras p21 protein activator 1 that inhibits the RAS-MAPK pathway. Inhibition of RASA1 by MEG3 activates the RAS-MAPK pathway and subsequently promotes the proliferation and invasion of trophoblasts.

## Methods

### Specimen collection

Recurrent spontaneous abortion refers to three or more consecutive spontaneous abortions. A total of 102 URSA patients (with gestational ages of 49–63 days) who were admitted to Shenzhen people’s hospital from January 2018 to October 2019 with the lack of cardiac pulsation of the primitive heart tube based on Color Doppler ultrasound participated in this study. Those patients were clinically diagnosed with missed abortions and had to undergo vacuum aspiration for terminating the pregnancy. The control group comprised 102 healthy and normal pregnant women of the same age group (with gestational ages of 49–63 days) who were admitted to our hospital during the same period to demand the elective termination of pregnancy via vacuum aspiration. Those pregnant women had no previous history of adverse pregnancy and had a history of one or more normal deliveries. They did not display the symptoms of threatened abortions, including vaginal bleeding. The color Doppler ultrasound examination revealed that their embryos normally developed (with embryos and cardiac pulsation of the heart tube, as well as embryonic sizes that are matched to the gestational ages).

Inclusion criteria: Research participants need to exclude endocrine, surgical and medical system diseases. There was no prethrombotic state and no deformity or inflammation of female reproductive system. The parental and embryonic chromosomes were normal. There were no autoantibodies such as antiphospholipid antibody, antinuclear antibody, anti DNA antibody, antisperm antibody and anti thyroid antibody. The male's semen was normal. There were no adverse living habits and hobbies such as strenuous exercise, taking caffeinated food and drinking during pregnancy.

The clinical data of all research participants was collected retrospectively from the medical record. The embryonic villis of all research participants were derived from tissue specimen bank of our department. Informed consents were obtained from all research participants and this study was approved by the ethics committee of Shenzhen people’s hospital.

### Establishment and culture of primary cell lines of villous trophoblast cells

Villis of 6 URSA patients and 6 normal pregnant women after induced abortion were obtained under aseptic conditions, and washed with PBS containing high concentrations of penicillin and streptomycin. Other substances such as decidua were cut off with ophthalmic scissors. The villi were picked out and finely chopped. Complex enzyme (containing 0.125% pancreatin, 4.2 mM MgSO_4_, 25 mM Hepes, and 20 U/mL DNaseI) was added for digestion for 20 min. The digestive juice was filtered, and the filtrate was collected and resuspended in 3 mL of serum-free DMEM. The cell suspension was slowly added to 15 mL centrifuge tubes coated with 60% and 35% Percoll solution in advance with a volume ratio of 1:1:1, and centrifuged for 20 min at 1500 rpm. The cloudy layer strip between the 60% and the 35% Percoll separation solutions was absorbed, centrifugally washed twice, and added to DMEM culture solution containing 10% calf serum for resuspension and culture.

Cell sorting was performed by flow cytometry (BD FACSVerse) to separate trophoblast cells with keratin 7 CK7( +) and vimentin VM(−) expression from the other cells. The cells obtained after sorting were identified by cell immunohistochemistry. The purity of trophoblast cells was measured as the ratio of CK7( +)VM(−) cells to total cells. The results showed that the purity of primary cytotrophoblast cells was > 99%, and URSA primary trophoblast cells (PT-U cells) and normal primary trophoblast cells (PT-N cells) were successfully cultured.

### Construction of cell lines that stably overexpress and underexpress MEG3

Lentiviral vectors harboring the MEG3 (MEG3 group) or the vector only (vector group) and lentiviral vectors harboring the MEG3-specific shRNA sequence (shRNA-MEG3 group) or the control sequence (shRNA-NC group) were purchased from GenePharma Co., Ltd. (Shanghai, China). Following transduction, the clones were selected with puromycin (1 μg/mL) to obtain PT-U cells wherein MEG3 was constitutively overexpressed (MEG3 group) and underexpressed (shRNA-MEG3), respectively. Sequences of shRNA-specific to the *MEG3* are included in Additional file 1: Table S1.

PT-U cell lines from 3 URSA patients were transfected with MEG3 or vector respectively, meanwhile PT-U cell lines from other 3 URSA patients were transfected with shRNA-MEG3 or shRNA-NC respectively. For there was no significant difference between the three cells lines in same group, the experimental data of three cells lines in same group was mixed for statistical analysis.

### Cell transfection

The full-length RASA1 sequence was cloned into pcDNA3.1 ( +) expression vector (GenePharma, Shanghai, China). The si-RASA1 were also purchased from GenePharma (Shanghai, China). Transfections were performed using a Lipofectamine 2000 kit (Invitrogen, Carlsbad, CA, USA) according to the standard instructions. Sequences of siRNA-specific to the RASA1 are included in Additional file 1: Table S1.

### RNA isolation and reverse transcription

The total RNA was extracted from PT-U cells and embryonic villi using the Trizol RNA extraction reagent (TaKaRa, Otsu, Japan), and reverse transcribed using the Prime-Script™ one-step RT-PCR kit (TaKaRa). The resulting cDNAs were used as a template for real-time quantitative PCR (qPCR) assay using SYBR Premix Ex Taq kit (TaKaRa), as per instructions provided in the kit, on the CFX96 Real-Time PCR Detection System (Bio-Rad, Hercules, California, USA). The relative expression level of *MEG3* was analyzed using the 2^−ΔΔCt^ method and normalized using *GAPDH* as the internal reference gene. The primer sequences are listed in Additional file 2: Table S2.

### Western blot assay

Cells grown to the log phase were harvested and lysed using the RIPA Lysis Buffer (Thermo Scientific, Belmont, Massachusetts, USA) and total proteins were extracted. An equal amount of protein (50 μg/lane) was separated on an SDS-PAGE and transferred onto a PVDF membrane (Roche, Basel, Switzerland), which was subsequently incubated overnight with the primary antibody (1:1000, Cell Signaling Technology, Massachusetts, USA) at 4 °C. After washing with PBST, the PVDF membrane was incubated with goat-anti-rabbit conjugated with HRP (1:2000; Santa Cruz Biotechnology; Dallas, TX, USA) at 25 °C for 1 h, followed by the addition of enhanced chemiluminescence (ECL) reagent (Thermo Scientific, Belmont, Massachusetts, US). Subsequently, the membrane was visualized and imaged on a gel imaging system. GAPDH was used as a loading control and detected by GAPDH antibody (1:1000, Cell Signaling Technology). The gray value of protein band was analyzed by using Quantity One scanning software (Bio-Rad, California, USA). The relative expression level of target protein = gray value of target protein band / gray value of GAPDH protein band.

### Cell proliferation assay

The cell proliferation assay was carried out using the Cell Counting Kit-8 (CCK-8) (Dojindo, Kumamoto, Japan). Briefly, cells grown to the log phase were seeded onto a 96-well plate at 1 × 10^3^ cells per well. After 0 h, 24 h, 48 h, and 72 h of seeding, 10 μL of CCK-8 working solution was added to each well, followed by a further incubation for 2 h prior to the measurement of absorbance at 450 nm (A_450_) using multifunctional microplate reader SpectraMax M5 (Molecular Devices, California, USA). The resulting A450 values were then used to plot the growth curve of cells.

### Apoptosis assay

The apoptosis assay was performed using the Annexin V-FITC Apoptosis Detection Kit (eBioscience, California, USA). Briefly, cells grown to the log phase (5 × 10^5^ cells) were detached from the plates with EDTA-free trypsin, washed with phosphate-buffered saline (PBS) and then centrifuged. The resulting cell pellet in each tube was mixed with 100 μL of binding buffer, 5 μL of annexin V-FITC, and propidium iodide (PI) and incubated in the dark at 25 °C for 20 min, followed by the determination of apoptosis using flow cytometry analysis.

### Cell invasion assay

A cell suspension of 1 × 10^6^ cells in a serum-free medium was added in the upper chamber of Transwell cell culture plate (coated with Matrigel, packaged with 24-well inserts, and has a pore size of 8-μm; Corning Costar, Cambridge, USA). The lower chamber contained the medium with 5% FBS. After 24 h of culture, the Transwell chambers were removed and fixed with methanol for 30 min. After washing the traces of methanol with PBS, the chambers were stained with 1% (v/v) crystal violet at room temperature for 20 min. Chambers were washed three times with PBS and cells that did not invade through the Matrigel in the upper chamber were wiped off carefully using a cotton swab, and the number of cells that invaded to the lower chamber were observed and counted under a light microscope.

### Embryo implantation assay

Healthy and sexually mature ICR female mice were selected. Dosages of PMSG (pregnant mare serum gonadotropin) (7.5 IU/mouse) and hCG (7.5 IU/mouse) were injected intraperitoneally in intervals of 46–48 h for superovulation. The female mice were caged with the male mice at a ratio of 1:1 on the day of hCG injection, and vaginal plugs were checked the next morning. Those with vaginal plugs were pregnant. After 96 h of hCG injection, the mice were sacrificed, and blastulae were removed. The blastulae were randomly divided into two groups. Those with the MEG3 and shRNA-MEG3 lentivirus infections formed the experimental groups, while those with the empty vector and the shRNA-NC lentivirus infection formed the control groups.

The decidua tissue of the mice was scraped under sterile conditions, placed in PBS containing two antibiotics (penicillin and streptomycin, Sigma, United States), cut to < 1 mm^3^, and digested with 0.1% type I collagenase (Sigma, United States) for 30 min. The cells were resuspended and inoculated in a cell culture dish at a density of 1 × 10^6^/mL. After subculture of the third generation, a single cell suspension was prepared with a cell density of about 7 × 10^4^/mL.

The blastulae cultured in vitro were transferred to a 12-well plate covered with decidual cells, by adding one blastula per well, and additionally 1 mL/well of implantation medium was added. The implantation process was observed under an inverted microscope, and after 72 h the number of implanted embryos was counted, and the implantation rates of the two groups were calculated. Embryo implantation was analysed the following way: when the culture plate was gently moved under the microscope, if the blastulae did not shake, it suggested that adhesion had taken place, and that the blastulae were fixed on the bottom of the wells and that adherence growth had started, and thus dissolution of surrounding decidual cells had occurred. After the implanted embryo count, the four groups of embryos were collected respectively, and the expression level of MEG3 was detected by RT-qPCR**.**

### Dual luciferase reporter assay

Cells were seeded into a 24-well plate at 5 × 10^4^ cells/well and incubated for 24 h in a humidified incubator. Cells were co-transfected with plasmids including, pcDNA-MEG3, pcDNA-EZH2, and the *RASA1*-WT dual luciferase reporter, as well as siRNAs for MEG3 (si-MEG3) and EZH2 (si-EZH2) (Genearray Biotechnology, China) using Lipofectamine 3000 (Invitrogen, Carlsbad, California, US), according to the manufacturer’s instructions. At 24 h post transfection, the luciferase activity in the cell lysate was determined using the Dual-Glo® Luciferase Assay System (Promega, Maddison, United States). Renilla luciferase activity was used to normalize the firefly luciferase activity.

### Chromatin immunoprecipitation assay (ChIP)

The ChIP assay was performed using the EZ ChIP Chromatin Immunoprecipitation Kit (Millipore, Bedford, MA, USA). Briefly, 1 × 10^7^ cells were cross-linked with 1% formaldehyde at room temperature for 10 min, followed by fragmentation of the chromatins to the size of 200–500 bp fragments using an ultrasonication. The DNA–protein complexes were immunoprecipitated using anti-EZH2 antibody, anti-H3K27me3 antibody, or anti-IgG antibody (1:100; Abcam, San Francisco, USA). The ChIP-derived DNA was subsequently amplified by qPCR method, and the percentage of input DNA was calculated. The Primers for the *RASA1* promoter are listed in Additional file 2: Table S2.

### RNA pulldown analysis

The RNA pulldown assay was performed using the Pierce Magnetic RNA Pull-Down Kit (Thermo Fisher Scientific, Waltham, MA, USA). Briefly, the MEG3 lncRNA was biotinylated (GenePharma, Shanghai, China) and incubated with the cell lysates. After that, the proteins bound with MEG3 lncRNA were captured by the M-280 streptavidin magnetic beads. Proteins that bound to MEG3 were isolated and subsequently identified by mass spectrometry and Western blot assay.

### DNA pulldown analysis

Primers designed to target the downstream of promoter region of *RASA1* gene were biotinylated (GenePharma) and incubated with the total RNA to allow the formation of DNA-RNA complexes, followed by the addition of Dynabeads MyOne Streptavidin C1 beads (Invitrogen) to pull down the DNA-RNA complexes using the biotinylated DNA. The co-purified RNA was subsequently treated with DNasel and subjected to the qPCR assay for determining the relative abundance of MEG3.

### RNA immunoprecipitation assay (RIP)

The RIP assay was performed using the EZMagna RIP-Kit (Millipore, Billerica, USA). Briefly, the cell lysate was incubated with EZH2 and IgG control antibodies overnight. The co-precipitated RNAs were then extracted and analyzed by qPCR method.

### Electrophoretic mobility shift assay (EMSA)

RNA EMSA was performed using LightShift Chemiluminescent RNA EMSA Kit (Thermo Fisher Scientific, Shanghai, China). Briefly, nuclear extracts were isolated from PT-U cells with NEPER Nuclear and Cytoplasmic Extraction Reagents (Thermo Fisher Scientific, Shanghai, China) according to the manufacturer’s instructions. Biotin-labeled RNA probes and unlabeled complementary RNA fragments were obtained by in vitro transcription assays with Biotin RNA Labeling Mix (Roche, Basel, Switzerland). Protein-lncRNA binding reactions were performed in proteins of PT-U cells along with Biotin-labeled RNA probes or unlabeled complementary RNA fragments. After incubation in 1 × EMSA binding buffer, the components were separated with native PAGE and then transferred on to positively charged nylon membrane (Roche, Mannheim, Germany). After UV cross-linking, biotin signals were detected with HRP–conjugated streptavidin according to the manufacturer’s instructions for the Chemiluminescent Nucleic Acid Detection Module (Thermo Fisher Scientific, Shanghai, China). For supershift analysis, 200 ng of anti-EZH2 antibody (Cell Signaling Technology, MA, USA) or IgG were added into the Protein–lncRNA binding reactions, followed by gel electrophoresis and ECL visualization.

### Statistical analyses

All statistical analyses in this study were performed using the SPSS 19.0 statistical software. All experiments were repeated at least three times, and the results were expressed as a mean ± standard deviation. The mean values between two groups were compared via independent samples t-test, while their correlations were analyzed using Pearson’s correlation coefficient. P < 0.05 indicates the statistically significant differences.

## Results

### MEG3 expression was low in embryonic villous tissues of URSA patients

**Fig. 1:**
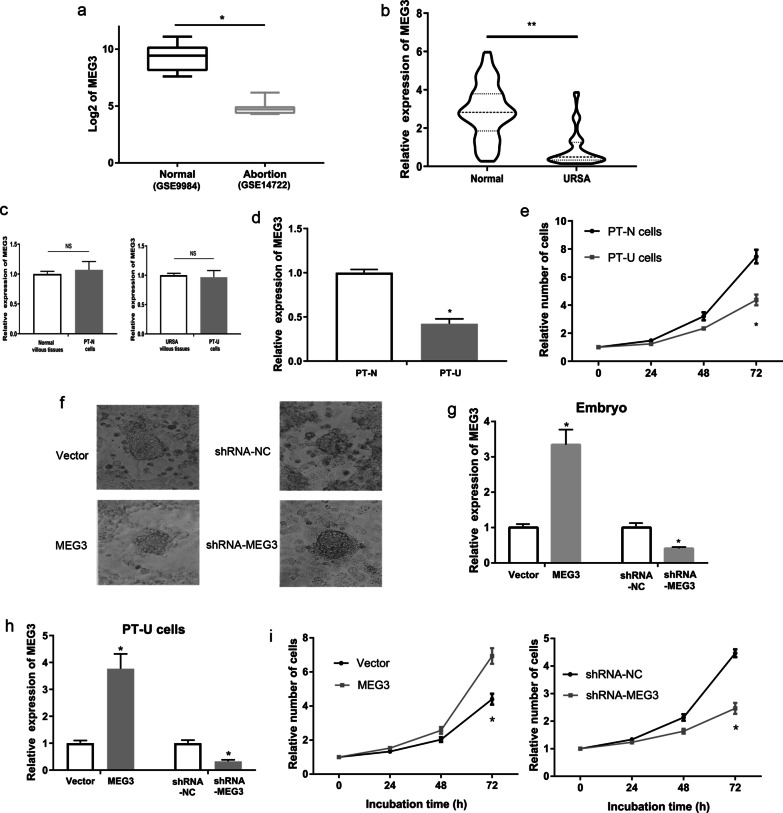
The expression and function of *MEG3* in trophoblast of URSA. **a** Analysis of RNA expression profile using the GSE14722 data (RNA-seq data of embryonic villi from 11 abortion patients) and GSE9984 data (RNA-seq data of embryonic villi from 12 normal pregnant women). **b** The expression levels of *MEG3* in embryonic villi from URSA patients and normal tissues using qRT-PCR assays. **c** Compare the expression of MEG3 in villi from URSA patients and PT-U cells (Primary trophoblast of URSA). **d** Compare the expression of MEG3 in PT-U cells and PT-N cells (normal primary trophoblast cells). **e** Compare the proliferative capacity of PT-U cells and PT-N cells. **f** The effect of MEG3 on the implantation capacity of mouse embryo in vitro. **g** The expression of MEG3 in MEG3 and shRNA-MEG3 transfected mouse embryo using qRT-PCR assays. **h** Effects of plasmids harboring *MEG3* sequence and shRNA-MEG3 on the expression level of MEG3 in PT-U cells using qRT-PCR. **i** The effects of MEG3 on the proliferative capacity of PT-U cells using CCK-8 assay. **P < 0.01, *P < 0.05

Database analysis of GSE14722 (RNA-seq data of embryonic villi from 11 abortion patients) and GSE9984 (RNA-seq data of embryonic villi from 12 normal pregnant women) shows that MEG3 expression was significantly lower in aborted villous tissues than in normal villous tissues (P < 0.01, Fig. 1a). Then we analyzed the expression of *MEG3* in embryonic villi from 102 URSA patients and the control group participants using qRT-PCR assay. The results showed that URSA patients had a significantly decreased level of MEG3 in embryonic villi when compared with that of the control group participants (P < 0.01, Fig. 1b).

Furthermore, Point-biserial correlation analysis showed that the expression level of MEG3 was negatively correlated with the URSA (Table1). Logistic regression analysis showed that low expression of MEG3 was a risk factor for URSA (Table1).

**Table 1:** The relationship between MEG3 and URSA

	N	MEG3 (mean ± SEM)	P-value	Point-biserial correlation	Odds ratio (95%CI)
NC	102	2.86 ± 0.14	< 0.001	− 0.609 (< 0.001)	0.319 (0.236–0.423)
URSA	102	0.97 ± 0.11			

### MEG3 promoted the implantation, proliferation, invasion and inhibited the apoptosis of PT-U cells

URSA primary trophoblast cells (PT-U cells) and normal primary trophoblast cells (PT-N cells) were used for cellular experiment. First, qRT-PCR assays showed that there was no significant difference of MEG3 expression between PT-U cells and villous tissues of URSA (Fig. 1c). Next, the expression of MEG3 significant decrease in PT-U cells compared with that in PT-N cells (P < 0.05, Fig. 1d). Last, CCK-8 assay indicated that PT-U cells had a significantly lower cell proliferative capacity when compared with PT-N cells (P < 0.05, Fig. 1e).

Furthermore, we evaluated the implantation ability of mice blastulae after treatment of MEG3 or shRNA-MEG3 vector. We transferred blastulae into 12 well plates with decidual cells and observed the number of implanted embryos 72 h later. The results showed that the implantation rate of embryos in the shRNA-MEG3 group at 72 h was 25% (18/71), and that of embryos in the shRNA-NC group at 72 h was 42% (30/71), with a significant difference between the two groups. The implantation rate of embryos was significantly increased in MEG3 group 81.9% (68/83), compared with that in control group 59.0% (49/83) (P < 0.05, Fig. 1f, Table 2, Table 3). At the same time, RT-qPCR showed that the expression of MEG3 in the embryonic tissue of the shRNA-MEG3 group was significantly lower than that in the shRNA-NC group. The expression of MEG3 in the embryonic tissue of the MEG3 group was significantly higher than that in the Vector group (P < 0.05, Fig. 1g).

**Table 2:** Embryo implantation rate of MEG3 group and Vector group

	Implantation		Implantation rate (%)	χ^2^ (P)
	−	+		
MEG3	15	68	81.9	9.381 (0.001)
Vector	34	49	59.0

**Table 3:** Embryo implantation rate of shRNA-MEG3 group and shRNA-NC group

	Implantation		Implantation rate (%)	χ^2^ (P)
	−	+		
shRNA-MEG3	52	25	32.5	12.605 (< 0.001)
shRNA-NC	29	48	62.3	

Subsequently, we generated PT-U cells that stably overexpressed or underexpressed MEG3, respectively. The results of qRT-PCR assays revealed that PT-U cells transduced with lentivirus harboring *MEG3* (MEG3 group) had a significantly higher level of MEG3 when compared with the control group (vector group), whereas PT-U cells transduced with lentivirus harboring shRNA-*MEG3* (shRNA-MEG3 group) had a significantly lower *MEG3* expression compared with the control group (shRNA-NC group) (P < 0.05, Fig. 1h). Furthermore, the results of CCK-8 assay for cell proliferation ability showed that the MEG3 group had a significantly higher cell proliferative capacity when compared with the control group, while the shRNA-MEG3 group had a significantly lower cell proliferative capacity when compared with the control group (P < 0.05, Fig. 1i).

**Fig. 2:**
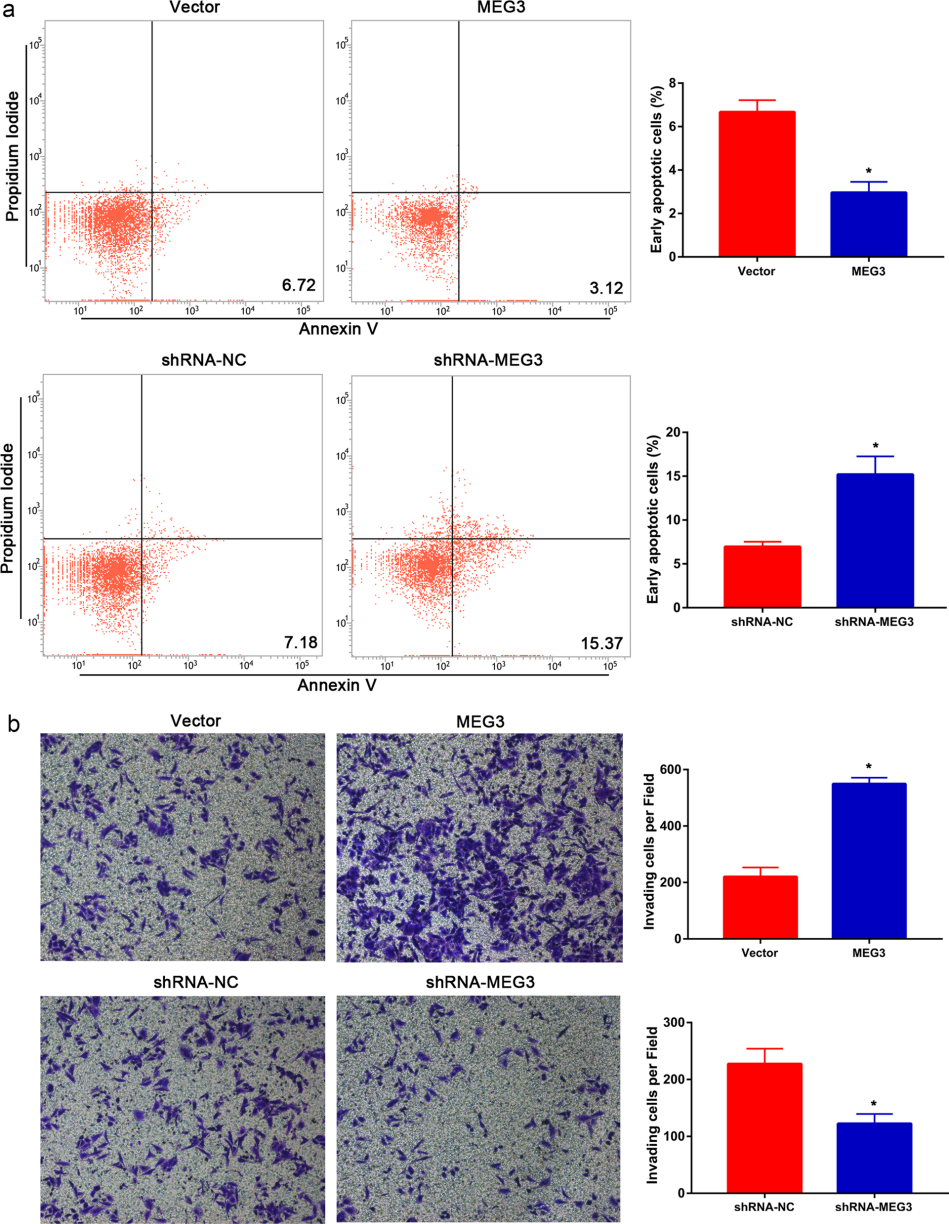
MEG3 inhibited the apoptosis and promoted the invasion of PT-U cells. **a** The effects of MEG3 on the apoptosis of PT-U cells using flow cytometry assay. **b** The effects of MEG3 on the invasive capacity of PT-U cells using Transwell invasion assay (*P < 0.05)

To study the apoptosis of PT-U cells, we performed flow cytometry analysis and showed that the MEG3 group had a significantly lower proportion of early apoptotic cells when compared with the control group. In contrast, the shRNA-MEG3 group had a significantly higher proportion of early apoptotic cells when compared with the control group (P < 0.05, Fig. 2a). Further, the results of Transwell invasion assay showed that the MEG3 group cells had a significantly higher invasive capacity compared to that of the control group, whereas the shRNA-MEG3 group cells had a significantly lower invasive capacity than that of the control group (P < 0.05, Fig. 2b).

### MEG3 inhibited the expression of *RASA1* and activated the RAS-MAPK pathway

**Fig. 3:**
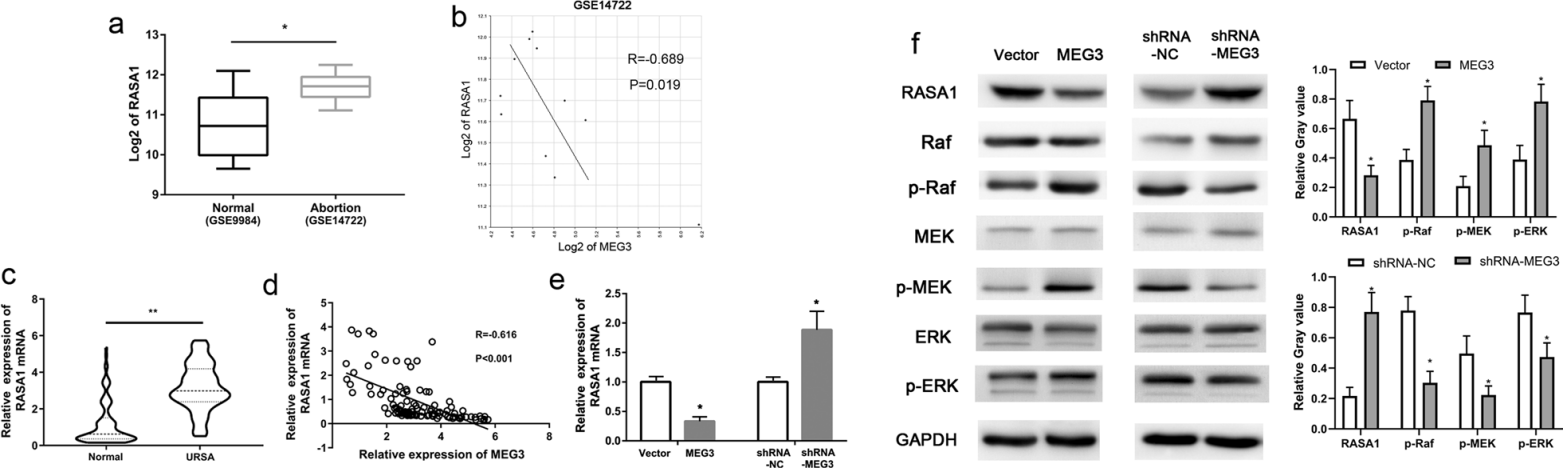
MEG3 inhibited the expression of RASA1 and activated the RAS-MAPK pathway. **a** and **b** Analysis of MEG3-associated proteins using the GSE14722 data and GSE9984 data. **c** The expression levels of *RASA1* in embryonic villi from URSA patients using qRT-PCR. **d** Correlation between the transcript levels of *MEG3* and *RASA1* in embryonic villi from URSA patients using qRT-PCR. **e** Effects of MEG3 on the transcript levels of *RASA1* in PT-U cells using qRT-PCR. **f** Western blot assay: Effects of MEG3 on the RASA1 protein levels in PT-U cells, as well as the levels of Raf, MEK, and ERK proteins (ratio of phosphorylated protein to total protein) in RAS-MAPK pathway. **P < 0.01, *P < 0.05

We analyzed the potential downstream targets of MEG3, which are associated with the proliferation and invasion pathways in trophoblasts using the GSE database. GSE14722 and GSE9984 data showed that *RASA1* gene (coding a protein that inhibits the RAS-MAPK pathway) transcription is significantly higher in aborted villous tissues than in normal villous tissues (P < 0.01, Fig. 3a). Correlation analysis on the GSE14722 revealed that the expression level of MEG3 had a significant negative correlation with the transcription of *RASA1* gene (R = − 0.616, P < 0.001, Fig. 3b). Next, we confirmed the transcript levels of *RASA1* gene in embryonic villi from 102 URSA patients and the control group participants using qRT-PCR assay. The results showed that URSA patients had significantly higher transcript levels of *RASA1* in embryonic villi than that of the control group participants (P < 0.05, Fig. 3c). The Pearson’s correlation analysis also showed that a significant negative correlation in the levels between the MEG3 and the *RASA1* mRNA in embryonic villi from URSA patients (R = − 0.738, P < 0.001, Fig. 3d). Therefore, we examined the expression level of *RASA1* gene in PT-U cells. Both qRT-PCR and Western blot assays revealed that the mRNA and protein levels of RASA1 were significantly reduced in the MEG3-overexpressing PT-U cells (MEG3 group) when compared to those in the control cells. On the contrary, the mRNA and protein levels of RASA1 significantly increased in the MEG3-downregulated cells (shRNA-MEG3) when compared to the control cells (P < 0.05, Fig. 3e, f). In addition, we then examined the activity of the RAS-MAPK pathway using Western blot assay. We showed that the levels of the phosphorylated Raf, MEK, and ERK protein significantly elevated in the MEG3-overexpressing PT-U cells (MEG3 group) when compared with those in the control group. However, the levels of the phosphorylated Raf, MEK, and ERK protein significantly deceased in MEG3- downregulated PT-U cells (shRNA-MEG3 group) when compared with those in the shRNA-NC group (Fig. 3f).

### EZH2 inhibited the *RASA1* gene transcription via histone methylation

**Fig. 4:**
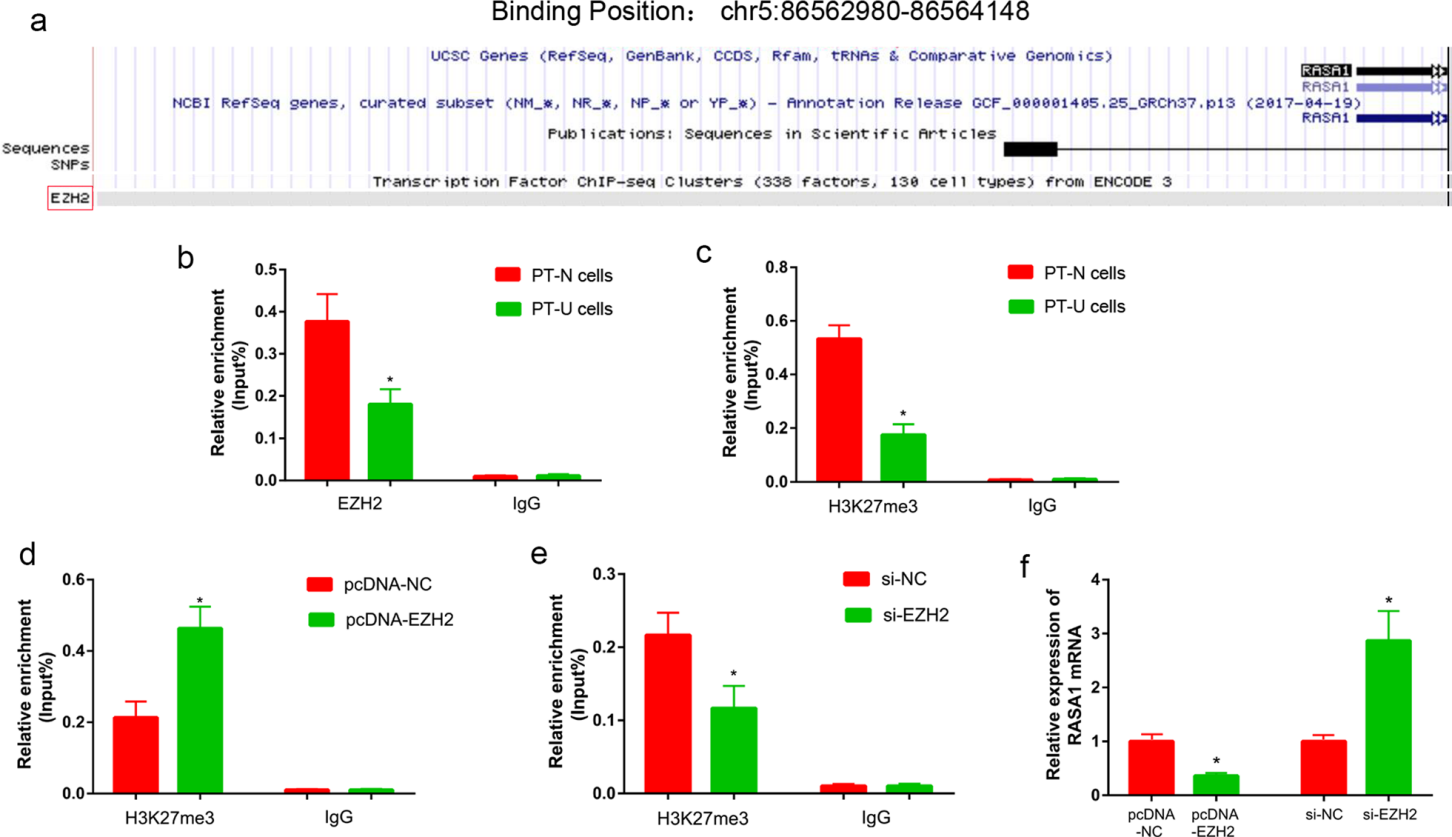
EZH2 regulated *RASA1* gene expression through histone methylation. **a** Bioinformatics analysis suggested presence of EZH2 protein binding site in *RASA1* gene promoter. ChIP assay was used to detect the enrichment of EZH2 (**b**) and H3K27me3 (**c**) in *RASA1* gene promoter of PT-U and PT-N cells. The enrichment of EZH2 in *RASA1* gene promoter of pcDNA-EZH2 (**d**) and si-EZH2 (**e**) transfected PT-U cells. **f** The expression of RASA1 in pcDNA-EZH2 and si-EZH2 transfected PT-U cells was detected by qRT-PCR

Bioinformatics analysis showed that there was a binding site of EZH2 in promoter region of the *RASA1* gene, from chr5: 86,562,980 to 86,564,148 (Fig. 4a). Then ChIP assay was used to analyze the enrichment of EZH2 and H3K27me3 at the promoter region of the *RASA1* gene in PT-U and PT-N cells. The results showed that there was less EZH2 enriched in promoter of the *RASA1* gene in PT-U cells than in PT-N cells (P < 0.05, Fig. 4b). H3K27me3 was also less enriched in the promoter of the *RASA1* gene in PT-U cells than in PT-N cells (P < 0.05, Fig. 4c). Further, the enrichment of H3K27me3 in promoter of *RASA1* gene was increased in the pcDNA-EZH2 transfected PT-U cells, compared with those in the control cells (P < 0.05, Fig. 4d). In contrast to this, the enrichment of H3K27me3 in promoter of the *RASA1* gene was decreased in the si-EZH2 transfected PT-U cells, compared with those in the control cells (P < 0.05, Fig. 4e). More important, the transcription level of *RASA1* gene was significantly down-regulated in PT-U cells of pcDNA-EZH2 group than those in control group. The transcription of *RASA1* gene was significantly up-regulated in PT-U cells of si-EZH2 group than those in control group (P < 0.05, Fig. 4f).

### MEG3 promoted histone methylation at the *RASA1* gene promoter

**Fig. 5:**
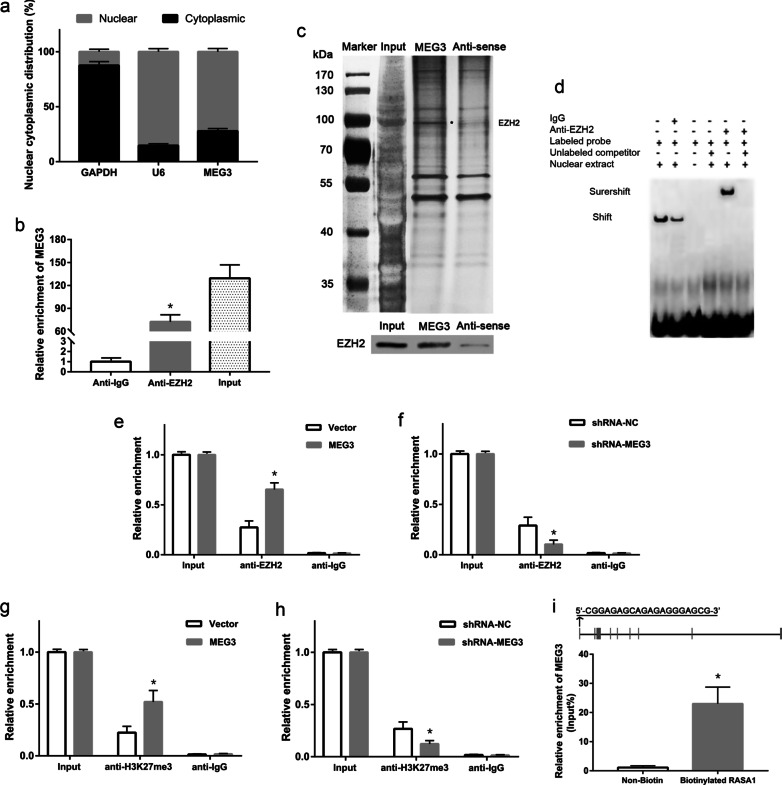
MEG3 regulated the expression of RASA1 via EZH2. **a** Cytoplasmic and nuclear localization of MEG3. **b** Determination of the ability of EZH2 protein to interact with MEG3 via RIP assay. **c** Identification of MEG3-bound proteins by RNA pulldown assay. **d** Verification the direct binding of EZH2 protein and MEG3 using EMSA assay. **e** and **f** ChIP assay for the recruitment of EZH2 at the *RASA1* promoter gene by MEG3. **g** and **h** Analysis of the effects of MEG3 on the levels of H3K27me3 at the *RASA1* promoter using ChIP assay. **i** GA-enriched motifs (chromatin-interacting sequences) at the 5’-end region of MEG3 sequence. Interactions of MEG3 with the promoter sequence of the *RASA1* gene using DNA pulldown assay. *P < 0.05

To gain insights in the underlying mechanism of MEG3 in the epigenetic regulation of *RASA1* expression, we analyzed the intracellular localization of MEG3 in PT-U cells and observed that MEG3 was mainly localized to nuclei (Fig. 5a). Next, we explored to identify epigenetics regulators that possibly bind to MEG3 lncRNA using an RNA pulldown assay. We demonstrated that MEG3 could bind to EZH2, which is a histone-lysine N-methyltransferase protein (Fig. 5c). Subsequent RIP assay showed that the EZH2 protein could enrich the MEG3 lncRNA in PT-U cells cells (P < 0.05, Fig. 5b). Moreover, EMSA assay also indicated the direct binding of EZH2 protein and MEG3 in PT-U cells (P < 0.05, Fig. 5d). Further, we analyzed the enrichment of EZH2 and H3K27me3 levels at the promoter region of *RASA1* gene using a ChIP assay. The results showed that the levels of EZH2 and H3K27me3 at the promoter region of *RASA1* gene were remarkably increased in the MEG3 group cells when compared with those in the control group cells. In contrast to this, the levels of EZH2 and H3K27me3 at the promoter region of *RASA1* gene significantly reduced in shRNA-MEG3 group cells when compared with the control group (P < 0.05, Fig. 5e–h). The sequence analysis of the MEG3 revealed the presence of chromatin-interacting sequences (GA-rich motifs) at the 5’-end region [Fig. 5i (Top)]. Further using DNA pulldown assay, we showed that compared to the control group, MEG3 lncRNA was significantly enriched at the promoter fragment of the *RASA1* gene [P < 0.05, Fig. 5i (bottom)].

### MEG3 inhibited the *RASA1* gene activity through EZH2

**Fig. 6:**
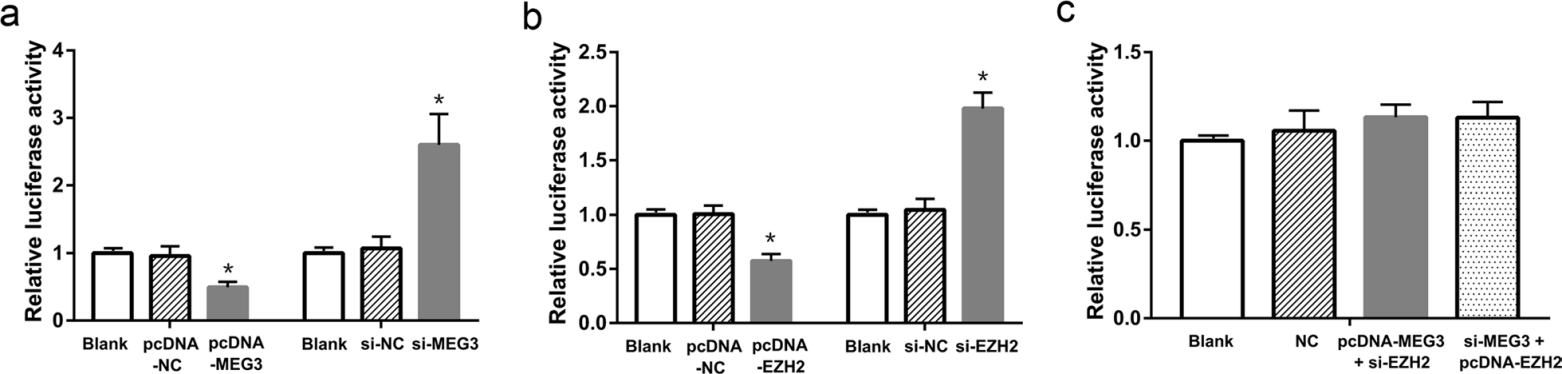
MEG3 decreased the RASA1 reporter activity via EZH2. Dual-luciferase reporter assay: **a** Effects of MEG3 on the luciferase activity of the *RASA1*-WT dual luciferase reporter. **b** Effects of EZH2 on the luciferase activity of the *RASA1*-WT dual luciferase reporter. **c** EZH2-mediated effects of MEG3 on the luciferase activity of the *RASA1*-WT dual luciferase reporter. *P < 0.05

Next, we co-transfected the cells with the *RASA1*-WT dual luciferase reporter plasmid and pcDNA-MEG3, pcDNA-NC (control group), or si-MEG3, si-NC (control group), or pcDNA-EZH2 (pcDNA-NC control group), or si-EZH2 and si-NC (control group). The results of dual luciferase reporter assay showed that cells co-transfected with *RASA1*-WT dual luciferase reporter plasmid and pcDNA-MEG3 had a significantly lower luciferase activity than that of the control group, while cells co-transfected with *RASA1*-WT dual luciferase reporter plasmid and si-MEG3 had a significantly higher luciferase activity than that of the control group (P < 0.05, Fig. 6a). Similarly, cells co-transfected with *RASA1*-WT dual luciferase reporter plasmid and pcDNA-EZH2 had a significantly lower luciferase activity than that of the control group, while cells co-transfected with *RASA1*-WT dual luciferase reporter plasmid and si-EZH2 had a significantly higher luciferase activity than that of the control group (P < 0.05, Fig. 6b). Meanwhile, we also compared cells co-transfected with *RASA1*-WT dual luciferase reporter plasmid + pcDNA-MEG3 + si-EZH2 and RASA1-WT dual luciferase reporter plasmid + si-MEG3 + pcDNA-EZH2, and found that there was no significant difference in the luciferase activity between the two groups, prompting the interference of MEG3-regulated RASA1 expression by EZH2 (P < 0.05, Fig. 6c).

### MEG3 regulated the functions of trophoblasts via RASA1

**Fig. 7:**
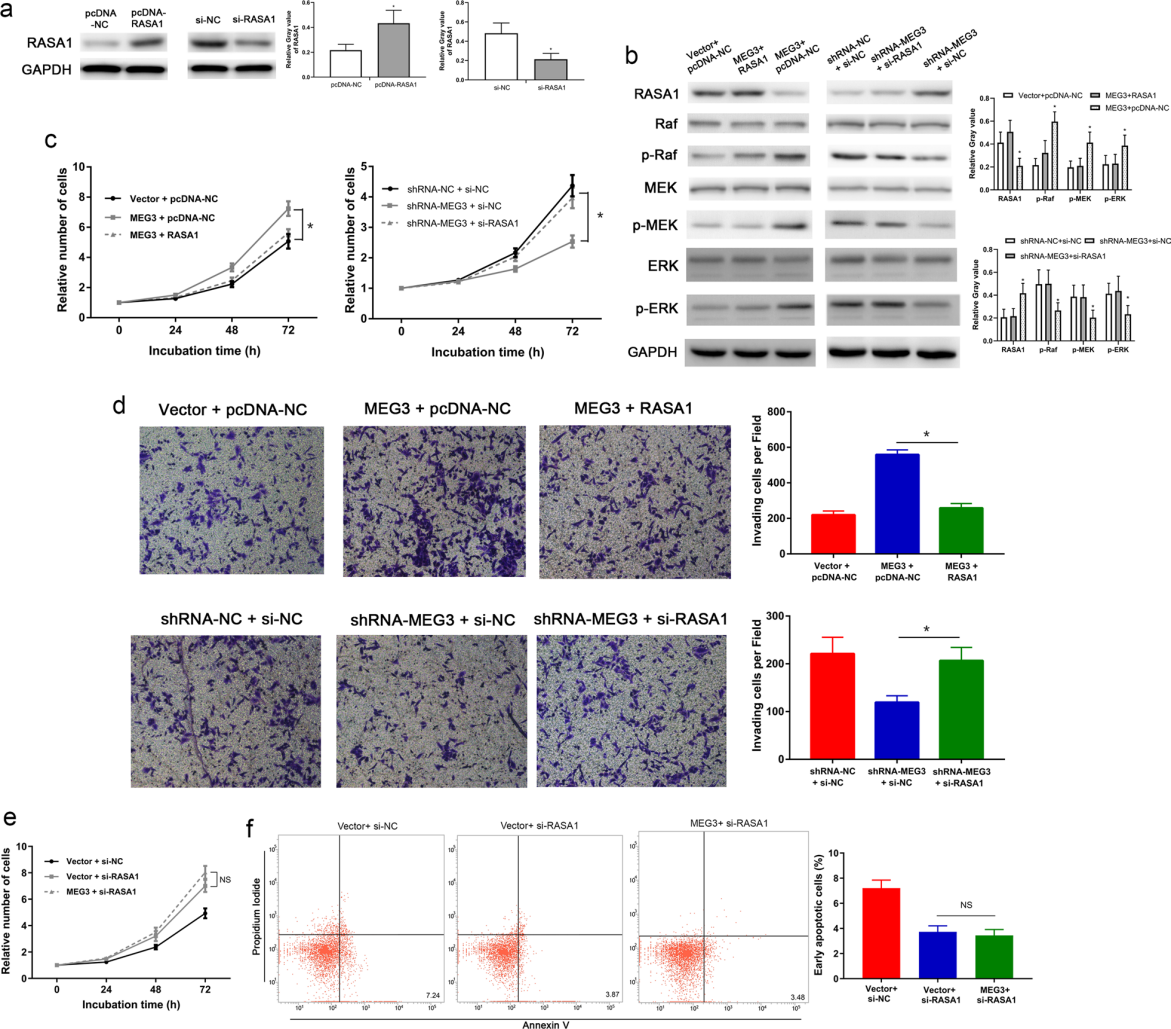
MEG3 modulated the RAS-MAPK pathway by regulating the expression of the *RASA1* gene. Western blot assay: **a** Effects of pcDNA-RASA1 and si-RASA1 on the RASA1 protein level in PT-U cells. **b** RASA1-mediated regulation in the activities of Raf, MEK, and ERK proteins in the RAS-MAPK pathway by MEG3. **c** The CCK-8 assay confirmed that RASA1 mediates the regulation of the proliferative capacity of PT-U cells by MEG3. **d** Transwell assay confirmed that RASA1 mediates the regulation of the invasive capacity of PT-U cells by MEG3. **e** and **f** CCK-8 and flow cytometry assay show that MEG3 has no effect when there is low RASA1 in PT-U cells (Vector + si-RASA1 V.S. MEG3 + si-RASA1). *P < 0.05

Our results with Western blot assay confirmed that pcDNA-RASA1 significantly increased the levels of RASA1 in PT-U cells, whereas si-RASA1 significantly reduced the level of RASA1 in PT-U cells (Fig. 7a). Subsequently, we reversed the inhibitory effect of MEG3 on RASA1 expression by transfecting pcDNA-RASA1 and the activating effect of shRNA-MEG3 on RASA1 expression by transfecting si-RASA1. Western blot assays were performed to validate reversal effects on the expression of RASA1 (Fig. 7b). The results showed that cells co-expressed with MEG3 and RASA1 had significantly lower levels of the phosphorylated Raf, MEK, and ERK proteins than the control group (co-transfected with MEG3 and vector control) (Fig. 7b). Our results with the CCK-8 assay for cell proliferation and Transwell invasion assays showed that cell proliferation and invasive capacities were significantly lower in cells expressing MEG3 and RASA1 than in the control group (Fig. 7c, d; P < 0.05 for both). Similarly, the Western blot assay also showed that cells co-transfected with shRNA-MEG3 + si-RASA1 had a significantly higher levels of the phosphorylated Raf, MEK, and ERK proteins when compared with the control group (co-transfected with shRNA-MEG3 + si-NC) (Fig. 7b). Additionally, the CCK-8 (P < 0.05, Fig. 7c) and Transwell (P < 0.05, Fig. 7d) assays further revealed significantly higher proliferative and invasive capacities of cells transfected with shRNA-MEG3 + si-RASA1 than that of the control group, respectively. Furthermore, we used si-RASA1 to suppress the expression of RASA1 protein and evaluated effect of MEG3 when there is low RASA1 in PT-U cells. CCK-8 and flow cytometry assay show that there was no difference between Vector + si-RASA1 group and MEG3 + si-RASA1 group (Fig. 7e, f). These results suggested that MEG3 regulates the RAS-MAPK pathway via RASA1, thereby affecting the proliferative, apoptotic, and invasive capacities of PT-U cells.

## Discussion

The association of the abnormal expression of lncRNAs in the regulation of gene expression during onset and disease progression has been extensively studied in the recent years (Zhou 2019; Horita 2019; Yu 2019). Some studies have also reported the relationship between lncRNAs and RSA. Wang et al. (2014) analyzed tissues of villi and decidua from patients with spontaneous and induced abortions using DNA microarray analysis and found that lncRNAs regulating infections and inflammations are the major pathogenicity factors leading to abortions. In addition, another study also reported that there were 1449 differentially expressed lncRNAs involved in 26 biological processes in placental villi of RSA patients. The functional analysis revealed that these lncRNAs are mainly involved in endocrine, immunity, apoptosis, and extracellular matrix (ECM)-receptor interaction (Wang et al. 2016). Both studies have analyzed the expression profiles of lncRNAs in placental villi from RSA patients, and the results indicated the close association between lncRNAs and the onset of RSA, thereby providing a theoretical basis for subsequent studies on the relationship between RSA and lncRNA, as well as the underlying mechanisms.

*MEG3*, a tumor suppressor gene, has been demonstrated to regulate the proliferation and invasion of various tumor cells. Our previous study have focused on the relationship between MEG3 and tumor cells function and its molecular mechanism. We found that MEG3 could inhibit the proliferation and invasion of some kind of tumor cells. In some case, MEG3 could regulate the activity of Wnt pathway. In other case, MEG3 might regulate the degradation of P-STAT3 protein (Zhang et al. 2016; Zhang et al. 2017a, b; Gao et al. 2017; Zhang et al. 2017a, b; Gao and Lu 2016; Zhang and Gao 2019).

In recent year, the roles of MEG3 in pregnancy-related diseases have started to gain attention among researchers. Zhang et al. (2015) found that MEG3 is expressed at a low level in placental tissues from patients with preeclampsia. Cell-based assays further confirmed that MEG3 leads to the migration and invasion of trophoblasts, as well as the remodeling of uterine spiral arteries, by affecting the expression of NF-κB, Caspase-3, and Bax proteins in trophoblasts, which results in the onset of preeclampsia. Similarly, Yu et al. (2018) found that the low expression of MEG3 in placental villi is closely associated with the onset of preeclampsia, whereby it affects the invasive capacity of trophoblasts by regulating the activity of TGF-β pathway. However, the involvement of MEG3 in the onset and progression of URSA remains unclear.

In this study, we found that *MEG3* was abnormally low expression in spontaneously aborted villous tissues using GSE RNA-seq data. We also confirmed that *MEG3* had a significantly low expression level in embryonic villi from URSA patients, suggesting that inactivation of *MEG3* may be involved in the onset and progression of URSA. This finding is consistent with the previous studies that revealed the low level of *MEG3* expression in placental villi from patients with other types of pathological pregnancies (Zhang et al. 2015; Yu et al. 2018). More important, logistic regression analysis suggested that the silent of *MEG3* maybe a risk fact for URSA. That mean MEG3 might have potential value in clinical practice of URSA.

Our subsequent investigations on the effects of MEG3 on the functions of trophoblasts showed that the inactivation of *MEG3* expression could inhibit the implantation, proliferation and invasion, as well as promote the apoptosis of trophoblasts, which is consistent with our findings that MEG3 was poorly expressed in embryonic villi from URSA patients. Abnormal embryo implantation is one of the important causes for URSA. Our embryo implantation assay confirmed that MEG3 has an important regulatory role in the implantation of mouse blastulae. These results strongly suggest that the abnormal expression of MEG3 is related to URSA. Thus, our results suggest that MEG3 might lead to the onset and progression of URSA by inhibiting the functions of trophoblasts.

To understand the mechanism of MEG3, we analyzed the GSE data of aborted villous tissues to search for the MEG3-related proteins that are involved in cell proliferation and invasion. We found that the RASA1 protein levels had a significant negative correlation with the MEG3 level, and *RASA1* gene may be a downstream target of MEG3. RASA1 is a key intracellular molecule and regulates signaling pathways associated with proliferation and apoptosis. RASA1 activates the intrinsic GTPase activity of Ras to promote the hydrolysis of Ras GTP to Ras GDP. RASA1 binds to activated Ras (Ras GTP) via the GTPase activating protein (GAP)-related catalytic domain to block the Ras signaling and inhibit the Ras-MAPK pathway, thereby inhibiting the proliferation but promoting the apoptosis of cells (Zeng 2019; Chen et al. 2019). Hence, we hypothesized that MEG3 activates the Ras-MAPK pathway by negatively regulating the protein levels of RASA1.

We validated the results obtained from the analysis of the GSE14722 data on tissues of embryonic villi from URSA patients and found that *RASA1* had a significantly high transcription level in those tissues and a significant negative correlation with the expression levels of MEG3, thus confirming the analysis of GSE14722 data. The subsequent cellular assays also confirmed that MEG3 had a negative regulatory effect on *RASA1* transcription and phosphorylation levels of the core proteins in the Ras-MAPK pathway. The results, as mentioned above, confirmed that MEG3 could regulate the Ras-MAPK pathway via RASA1 protein.

We further investigated the molecular mechanism of MEG3 in the regulation of RASA1. Our analysis on the localization of MEG3 in trophoblasts revealed that MEG3 was predominantly localized to nuclei. MEG3 could inhibit the mRNA levels of the *RASA1* gene. These data indicated that MEG3 might regulate the expression of *RASA1* at the transcriptional level. Further more, bioinformatics analysis suggested the presence of EZH2 binding site in the promoter of *RASA1* gene. The EZH2 RIP-seq carried out by Wang et al. (2018) on organ and muscle tissues from mice uncovered a total of 1328 lncRNAs (including MEG3) that could bind to EZH2 protein. Kaneko et al. (2014) found that MEG3 could epigenetically regulate the expression of its target genes by binding to the Polycomb repressive complex-2 (PRC2, which comprises EZH2 as its core protein subunit). Based on these studies, we speculate that MEG3 might epigenetically regulate the expression of *RASA1* through EZH2.

So we first proved that EZH2 was enriched in promoter of *RASA1* gene and could regulated the transcription of *RASA1* gene in trophoblasts. Then we found the binding between MEG3 and EZH2 in trophoblasts using an RNA pulldown and RIP assays. Our ChIP assay confirmed that MEG3 could recruit EZH2 at the promoter of the *RASA1* gene, which resulted in an increase of H3K27me3 level at the *RASA1* promoter. The dual luciferase reporter assay also demonstrated that MEG3 and EZH2 could inhibit the activation of *RASA1*, and the inhibitory effect of MEG3 on RASA1 could be reversed upon knockdown of EZH2, suggesting that both MEG3 and EZH2 regulate the expression of RASA1 via the same pathway.

Current studies indicated that some lncRNAs harbor GA-enriched motifs that can facilitate their localization to chromosomes and directly regulate the target genes expression by forming RNA–DNA triplexes (Chu et al. 2011; Mondal 2015). It remains unclear whether the direct regulation of *RASA1* expression by MEG3 is achieved via the recruitment of EZH2 to the promoter region of the *RASA1* gene. Our bioinformatics analysis on the MEG3 sequence revealed the presence of GA-enriched motifs at the 5’-end region, which suggests that MEG3 does exhibit the biological feature for binding to chromosomes. In DNA pulldown assay, we showed that MEG3 could bind to the promoter sequence of the *RASA1* gene, thereby further demonstrating that MEG3 facilitates the epigenetic regulation of *RASA1* by recruiting EZH2 to the promoter region of the *RASA1* gene.

Lastly, we investigated whether MEG3 modulates the functions of trophoblasts by regulating RASA1 and the RAS-MAPK pathway. We used si-RASA1 to down-regulated RASA1 expression and found that when RASA1 protein was low expressed, the biological effects of MEG3 were greatly weakened in trophoblasts. The activating effect of MEG3 on the core proteins in the RAS-MAPK pathway, as well as the proliferative and invasive capacities of trophoblasts, reduced after attenuating the regulation of RASA1 by MEG3 in trophoblasts. The above results demonstrated that MEG3 modulates the functions of trophoblasts by regulating RASA1 and RAS-MAPK pathway.

## Conclusions

In summary, our study has confirmed the association of *MEG3* inactivation, in embryonic villi, with URSA. MEG3 leads to the silencing of *RASA1* and the activation of the RAS-MAPK pathway in trophoblasts by recruiting EZH2 to the promoter region of the *RASA1* gene, thereby enhancing the implantation, proliferative and invasive capacities of trophoblasts.

## Supplementary Information

**Additional file 1: Table S1.** Oligonucleotide sequence.

**Additional file 2: Table S2.** PCR primers.

## Data Availability

All data generated or analyzed during this study are included either in this article or in the additional files.

## Abbreviations

ChIP: Chromatin immunoprecipitation
EMT: Epithelial-mesenchymal transition
ERK: Extracellular-signal-regulated kinase
GSE: Genomic spatial event
lncRNA: Long noncoding RNA
MEG3: Maternally expressed gene 3
miRNAs: MicroRNAs
MEK: Mitogen-activated protein kinase kinase
Raf: Rapidly accelerated fibrosarcoma
RASA1: RAS P21 protein activator 1
RIP: RNA immunoprecipitation
ROC: Receiver operator characteristic curve
shRNA: Short hairpin RNA
URSA: Unexplained recurrent spontaneous abortion
