# Supplementary material for: The long noncoding RNA MEG3 regulates Ras-MAPK pathway through RASA1 in trophoblast and is associated with unexplained recurrent spontaneous abortion
Source: Mol Med. 2021 Jul 8;27:70. doi: 10.1186/s10020-021-00337-9 (PMC8265043; doi:10.1186/s10020-021-00337-9)
Supplement: Supplementary file 1 — Additional file 1: Table S1. Oligonucleotide sequence. [file 10020_2021_337_MOESM1_ESM.doc]

Table S1 Oligonucleotide sequence

| Name | Sequence |
| --- | --- |
| MEG3 shRNA | 5'-GGTTGTTGTGAGAATTAAA-3' (forward)  5'-TTTAATTCTCACAACAACC-3' (reverse) |
| NC shRNA | 5'-UAAUCCGAACGUGUCACGUTT-3' (forward)  5'-ACGUGACACGUUCGGAGAATT-3' (reverse) |
| si-RASA1 | 5’- CAUAGAUCACUAUCGAAAATT-3’ (forward)  5’- UUUUCGAUAGUGAUCUAUGAT-3’ (reverse) |
