# Supplementary material for: The long noncoding RNA MEG3 regulates Ras-MAPK pathway through RASA1 in trophoblast and is associated with unexplained recurrent spontaneous abortion
Source: Mol Med. 2021 Jul 8;27:70. doi: 10.1186/s10020-021-00337-9 (PMC8265043; doi:10.1186/s10020-021-00337-9)
Supplement: Supplementary file 2 — Additional file 2: Table S2. PCR primers. [file 10020_2021_337_MOESM2_ESM.doc]

Table S2 PCR primers

| Gene Name | Forward Primer (5’-3’) | Reverse Primer(5’-3’) |
| --- | --- | --- |
| MEG3 | 5′- CATCCGTCCACCTCCTTGTCTTC -3′ | 5′- GTCCTCTTCATCCTTTGCCATCC -3′ |
| GAPDH | 5′-TGCACCACCAACTGCTTAGC-3′ | 5′-GGCATGCACTGTGGTCATGAG-3′ |
| RASA1 for ChIP | 5′-CCCGCCTTCCTTTCTCTCTC -3′ | 5′- CGAAAACTTCCTCGCTGCAC -3′ |
